# Ultrasound-driven co-culture fermentation enhances bioactive components, antioxidant activity and structural properties of Chinese yam (*Dioscorea opposita Thunb*)^☆^

**DOI:** 10.1016/j.ultsonch.2026.107882

**Published:** 2026-05-22

**Authors:** Aysha Imtiaz, Sanabil Yaqoob, Kanza Aziz Awan, Hiba Naveed, Ahmad Faraz, Waleed Sultan, Remah Sobhy, Akmal Nazir, Jian-Ya Qian, Qing Shen

**Affiliations:** a School of Food Science and Engineering, Yangzhou University, Yangzhou 225127, Jiangsu, China; b Department of Food Science & Bioengineering, Zhejiang Gongshang University, Hangzhou 310018, China; c Panvascular Diseases Research Center, The Quzhou Affiliaed Hospital of Wenzhou Medical University, Quzhou People’s Hospital, Quzhou 324000, China; d Laboratory of Food Nutrition and Clinical Research, Institute of Seafood, Zhejiang Gongshang University, Hangzhou 310012, China; e Department of Food and Nutritional Sciences, Faculty of Science and Technology, University of Central Punjab, Lahore, Pakistan; f Biochemistry Department, Faculty of Agriculture, Benha University, Qalubia 13736, Egypt; g School of Food and Biological Engineering, Jiangsu University, Zhenjiang, China; h Department of Food Science, College of Agriculture and Veterinary Medicine, United Arab Emirates University, 15551 Al Ain, United Arab Emirates

**Keywords:** Chinese yam, Ultrasonication, Fermentation, Antioxidants, Sensory, Structural analysis

## Abstract

This study investigates the impact of multi-frequency ultrasound-assisted fermentation using *Lactobacillus plantarum*, *Lactobacillus paracasei* and *Lactobacillus helveticus* on the bioactive moieties composition, antioxidant assay and structural properties of Chinese yam (*Dioscorea opposita* Thunb). Ultrasound treatment improved microbial activity and mass transfer, promoting the release and biotransformation of bound phenolic components. As a result, total phenolic content increased from 689.36 to 8109.74 µg/mL, while total flavonoid content rose from 110.67 to 1158.48 µg/mL in the optimized treatment (CY_6_). Antioxidant potential exhibited notable enhancement, with ferric reducing antioxidant power (FRAP) increasing from 651.60 to 1337.14 µmol/L and DPPH radical scavenging activity revealing a moderate increase (values expressed as % inhibition; calculation method specified). HPLC quantification presented substantial increases in key phenolics, such as quinic acid (228.94 to 2912.46 µg/mL) and catechin (25.58 to 66.62 µg/mL). Structural analysis (SEM and XRD) manifested reduced crystallinity and increased porosity, which may facilitate the release of bioactive components. Sensory profiling demonstrated improved flavor characteristics, particularly enhanced floral and fruity notes in fermented treatments. Overall, ultrasound-assisted LAB co-culture fermentation boosted the physicochemical and functional attributes of Chinese yam, suggesting its potential for development as a value-added functional ingredient.

## Graphical abstract

**Figure:**
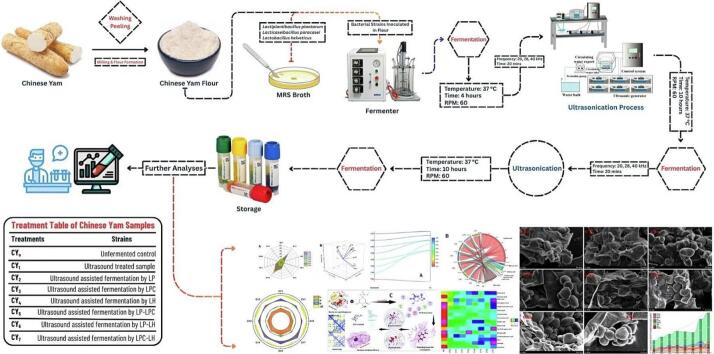

## 1 Introduction

The *Dioscoreaceae* family composed of over 600 species of tuberous perennial plants, recognized particularly for their ecological adaptability and pharmacological use in ancient times. Among these, *Dioscorea opposita Thunb*., which is commonly known as Chinese yam holds particular significance in East Asian countries, particularly China, Korea and Japan due to its rich nutritional profile and medicinal effects such as antioxidant, anti-inflammatory and immunomodulatory impacts [1]. Recent researches on Chinese yam contains a wide range of bioactive components including phenolic acids, flavonoids, saponins, mucilage polysaccharides, allantoin and diosgenin. These phytoceutical substances are linked with multiple therapeutic effects such as antioxidant, anti-inflammatory, antidiabetic and immunomodulatory activities. Oxidative stress due to free radicals production is recognized as a major contributing factor in the development of chronic illnesses and age-related disorders such as cardiovascular disease, diabetes, cancer and neurodegenerative conditions [2]. Despite the nutritional profile of Chinese yam, the bioavailability and stability of its bioactive moieties can be limited due to the structural changes within the tuber matrix. This highlights the need for effective processing techniques to improve the release and functionality of these compounds. Oxidative stress leads to cellular and molecular damage by disrupting the balance between reactive oxygen species production and the antioxidant defense system of the body, ultimately promoting disease progression and tissue dysfunction. Chinese yam’s rich phytochemical composition highlights its strong potential as a natural antioxidant source, warranting further exploration of its bioactive compounds and structural properties. Moreover, the conventional fermentation methods have been investigated to improve the functionality as well as the stability of yam. However, a novel way to get over these limitations is to combine lactic acid bacteria (LAB) fermentation with multi-frequency ultrasound [3].

Although the recent years have witnessed increasing research to Chinese yam as a functional commodity, it should be acknowledged that there is a dearth of information related to the full assessment of the antioxidant properties of this product, as well as the complexity of its photo-metric profile. The underutilized secondary metabolites of Chinese yam including acylated flavonoids, phenolic conjugates and peptide compounds make up about 40% of the plant's total secondary metabolites, so presenting a significant limitation in current research. The latest developments in analysis have made it possible to determine the chemical makeup of Chinese yam, whereas using a combined method of multi-frequency ultrasound (20/28/40 kHz) along with LAB fermentation improves the metabolic, structural and sensory characteristics, resulting in a consistent way of improving nutrition and sensory quality [4]. Previous studies on Chinese yam processing have primarily focused on either conventional fermentation or ultrasound treatment applied independently, with limited attention to their combined effects on bioactive moities release, structural alterations and sensory profiling. Moreover, the synergistic impact of multi-frequency ultrasonication and LAB co-culture fermentation remains insufficiently explored, particularly in relation to simultaneous optimization of physicochemical, functional and microstructural chacaterization [5].

Another research by Yaqoob, Imtiaz, Khalifa, Maqsood, Ullah, Shahat, Al-Asmari, Murtaza, Qian and Ma [3], elucidates the multi-frequency ultrasound (20/28/40 kHz) was integrated with LAB co-culture fermentation (*Lactobacillus plantarum*, *Lactobacillus paracasei* and *Lactobacillus helveticus*) to investigate their combined effects on Chinese yam flour. These results offer new insights in form of novel experimental observations indicating improvement in the liberation and conversion of bioactive compounds with an evident increase in the amounts of phenolics, flavonoids and certain phenolic acids. Moreover, structural studies revealed that there is decreased crystallinity and increased porosity. Furthermore, sensory evaluation showed marked differences in the flavor profile, especially floral and fruity characteristics. Ultrasonication at multiple frequencies provides acoustic cavitation, which causes localized shear stress leading to cell wall disruption and enhancing mass transfer [6]. These effects, when used in combination with microbial fermentation, lead to the release of the bound phytochemicals and enable the enzyme mediated biotransformation of substrates facilitated by LAB. Such integration of technologies allows an improved ability to modify the food matrix when compared to either treatment separately used in previous research works. To conclude, this study focuses on the critical issue by conducting the systematic assessment of the synergic effects of ultrasonic-assisted co-culture fermentation using Chinese yam [7].

Moreover, the technology is effective in improving the sensory attributes through precise manipulation of the microstructure and texture aspects, together with functional bioavailability through controlled manipulation of the food matrix in order to enhance the release of nutrients. Our comprehensive research provides mechanistic insight into the interaction between the physical effects of ultrasonic technology and the metabolic activities of microbes.

## 2 Materials and methods

### 2.1 Procurement of raw material

The mature roots of the *Dioscorea opposita* were obtained from local markets in Yangzhou, Jiangsu Province, China. The selection criterion included a consistent root size and lack of deformities. Following acquisition, the roots were preserved in a refrigerator at 4°C for processing within 48 h. The tubers were thoroughly washed, peeled and stored at −18°C until further processing. The peeled tubers were sliced into uniform pieces (approximately 3–5 mm thickness) to ensure consistent drying and processing. Prior to freezing, the moisture content of fresh yam was around 70–75%, as typical for tuber crops. Subsequently, they were milled into flour using a laboratory-scale milling machine. The obtained flour was sieved (e.g., 60-mesh) and stored in airtight containers at ambient conditions (25 ± 2°C) until further analysis to prevent moisture absorption. *Lactiplantibacillus plantarum* ATCC 8014, *Lacticaseibacillus casei* ATCC 334 and *Lactobacillus helveticus* ATCC 15009 were obtained from SynBio Tech (Beijing, China). All strains were cultured in de Man, Rogosa and Sharpe (MRS) medium at 37°C and stored at 4°C until use. All chemicals and solvents used were of analytical grade.

### 2.2 Multifrequency ultrasonication-assisted fermentation conditions

The fermentation process of Chinese yam flour using multifrequency ultrasound was done based on the optimized condition. The three probiotics including Lactiplantibacillus plantarum, *Lacticaseibacillus paracasei* and *Lactobacillus helveticus* were grown in MRS broth in an HZQ-F160 incubator up to the log phase (10^7^–10^8^ CFU/mL). The power intensity of ultrasound-assisted fermentation is 400 W, amplitude of 60% and the sample volume upto100 mL per batch, time for the treatment is 20 min the temperature was controlled and maintained at 25 ± 2°C using an ice bath to prevent thermal degradation Probe-type ultrasonicator (20 kHz) with titanium probe (diameter: 13 mm). The method causes the cavitation, which helps improve cell wall breakdown, thereby facilitating the release of intracellular bioactive substances. This choice of lactic acid bacteria is grounded in their demonstrated capabilities from the standpoint of both functionality and technology when used in fermentations involving plant ingredients. *L. plantarum* is noted for good adaption to plant matrices as well as for possessing abilities to acidify plant materials and increase bioavailability of phenolics through enzymes like β-glucosidase. *L. paracasei* is valuable from a perspective of probiotic capability, protein degradation and sensory benefits. Finally, *L. helveticus* is characterized by efficient protein degradation and production of biologically active peptides. It should be noted that this combination may also have some synergistic effects [8]. A 2% (v/w) bacterial inoculum was aseptically introduced to sterilized yam flour, followed by primary fermentation at the temperature of 37°C with orbital agitation (60 rpm) for 4 h. Afterwards, the ultrasonication was conducted using a hexagonal bath operating in tri-frequency mode (20/28/40 kHz) at 50 W/L power density with intermittent pulses (10 sec on/10 sec off) for 20 min total exposure while maintaining temperature at 25 ± 2°C *via* water circulation. The yam substrate was milled to flour after cleaning and subjected to freezing at −18°C. After sonication treatment, the samples were subjected to second fermentation for 10 h, thereby completing a 24-hour cycle of fermentation and sonication treatment [9]. The samples' codes are shown in Table 1.

**Table 1:** Treatment table of Chinese yam samples.

**Treatments**	**Strains**
CY_o_	Unfermented control
CY_1_	Ultrasound treated sample
CY_2_	Ultrasound assisted fermentation by *Lactiplantibacillus plantarum*
CY_3_	Ultrasound assisted fermentation by *Lactiplantibacillus casei*
CY_4_	Ultrasound assisted fermentation by *Lactobacillus helveticus*
CY_5_	Ultrasound assisted fermentation by *Lactiplantibacillus plantarum* and *Lactiplantibacillus casei*
CY_6_	Ultrasound assisted fermentation by *Lactiplantibacillus plantarum* and *Lactobacillus helveticus*
CY_7_	Ultrasound assisted fermentation by *Lactiplantibacillus casei* and *Lactobacillus helveticus*

### 2.3 Quantitative and qualitative analysis of the extracted polyphenols

To extract the free phenolic compounds in the sample, the method prescribed by El-Hadary [10] was employed. 1 g of the sample was extracted with 20 mL of 80% (v/v) methanol, followed by sonication treatment for a period of 30 min and agitation speed at 150 rpm. Subsequently, the centrifugation was done at 4000 g for 10 min and the resulting supernatant was lyophilized and preserved as the undigested fraction for analyses. This solvent system was selected due to its high polarity, which effectively solubilizes a broad range of phenolic components such as flavonoids and phenolic acids. Prior studies revealed that methanol–water mixtures provide higher extraction efficiency compared to pure solvents. The chosen method ensures improved recovery of total phenolics due to enhanced solvent penetration and mass transfer [11], [12].

The total flavonoid content (TFC) total phenolic content (TPC) of the samples was determined from the sample using the methods outlined by Chen, Chen, Xiao and Fu [13]. A mixture containing 100 μL of ddH_2_O, 10 μL of 5% sodium nitrite solution and 25 μL of the sample was allowed to stand for 5 min. Thereafter, 50 μL of sodium hydroxide and ddH_2_O alongside 15 μL of 10% AlCl_3_ were added. Measurements of absorbance were done at 510 nm. The determination of TFC was done using quercetin as a standard and the results were expressed as mg of rutin equivalents (mg RE) per mL of sample. A reaction mixture was made up using 1 mL Folin-Ciocalteau reagent, 3 mL 20% w/v Na_2_CO_3_ solution, 12 mL ddH_2_O and 200 μL sample. The mixture was allowed to react at 70°C in water bath for 10 min. Absorbance was read at 765 nm using a SpectraMax i3 spectrophotometer (Molecular Devices, Silicon Valley, CA, USA). TPC was calculated based on a gallic acid standard curve and results were expressed as mg of gallic acid equivalents (mg GAE) per mL of sample.

The total flavonols content Tflav of Chinese yam samples was determined by the procedure of Yaqoob, Imtiaz, Khalifa, Maqsood, Ullah, Shahat, Al-Asmari, Murtaza, Qian and Ma [3] with slight modifications. Shortly, a 2 mL sample, 2 mL AlCl_3 and_ 3 mL CH_3_COONa were mixed and stored at 20°C for 15 min. Finally, the absorbance was recorded at 440 nm using a SpectraMax i3 spectrophotometer. The Tflav were calculated using quercetin as a standard and results were represented in mg of quercetin equivalent/mL of sample.

Proanthocyanidin concentration (PAC) was measured using the method by Esquivel-Alvarado, Muñoz-Arrieta, Alfaro-Viquez, Madrigal-Carballo, Krueger and Reed [14], which involves mixing the sample with concentrated HCl and vanillin reagent. A volume of 1 mL of the sample was mixed with 3 mL of concentrated hydrochloric acid and 6 mL of vanillin solution. This was followed by an incubation period of 15 min at 25°C. The absorbance was taken at 500 nm. The polyphenols content was determined using a catechin calibration curve expressed in mg of catechin per mL.

Eventually, polyphenols were evaluated using an Agilent 1260 Infinity II HPLC-UV system, following the method of Dou, Chen, Fu and Liu [15] with minor changes. Separation was achieved using a C18 column (Agilent Zorbax SB, 4.6 × 250  mm, 5  µm particle size). The mobile phase consisted of Solvent A (0.1% acetic acid in water) and Solvent B (100% acetonitrile) in a linear gradient mode of 5 to 10% B (0 to 10 min), 10 to 15 min, 10 to 20% B; 15 to 25 min, 20 to 38% B; 25 to 30 min, 38 to 40% B; 30 to 31 min, 40 to 100% B; 31 to 35 min, 100% B; 35 to 36 min. Detection was carried out at 250  nm for general phenolics and 284  nm for flavonoids. The concentrations were calculated using calibration curves based on peak regions, alongside identification and quantification were carried out by comparing the retention durations and UV spectra with those of verified standards.

### 2.4 Evaluation of the antioxidant activity

The sample was evaluated for 2,2-diphenyl-1-picrylhydrazyl free radical scavenging activity using the methodology described by El-Hadary [16]. A volume of 120 µL of the sample was combined with 4.2 mL of the methanolic solution containing DPPH with a concentration of 0.1 mM. The mixture was then allowed to rest in the dark for 30 min. The absorbance was finally measured using the UV–Vis spectrophotometer at 517 nm wavelength. To calculate the radical scavenging activity, the following formula was used:

(1) $$DPPH\;scavenging\;activity\;\left(\%\right)=\;Control-\;sample/control\times 100$$

The FRAP of the sample was evaluated following Hsieh and Rajashekaraiah [17]. FRAP reagent was made by adding 25 mL of acetate buffer (0.3 M, pH 3.6), 2.5 mL of 10 mM TPTZ solution in 40 mM HCl and 2.5 mL of 20 mM FeCl_3_ · 6H_2_O. For the assay formation, 0.3 mL of FRAP reagent was added to 1.0 mL of the sample and 0.3 mL of ddH_2_O. The final reaction mixture was placed in a 37°C water bath for 30 min. Absorbance was then recorded at 595 nm by utilizing a UV–Vis spectrophotometer and results were expressed as mg of Trolox equivalents (mg TE/mL).

There were three main steps involved: (i) primary fermentation (4 h at 37 degrees Celsius) to trigger biological action and conversion of substrate material; (ii) multifrequency ultrasonication (20 min, 20/28/40 kHz) that acted as an intensifier to boost mass transfer and break down cellular structure while facilitating interaction between enzymes and substrates; and (iii) secondary fermentation (10 h at 37 degrees Celsius) for more biological conversion of newly released compounds.

Moreover, the H_2_O_2_ scavenging activity of yam was calculated by utilizing the methodology described by Inavally, D’Souza and Sadananda [18] with slight changes. A 2  mL sample at various concentrations was mixed with 2.0  mL of H_2_O_2_ (40  mM) prepared in phosphate buffer (pH 7.4). Following this, the mixture of the reaction was then incubated at room temperature for 10 min. UV–Visible spectrophotometry was employed to determine the absorption value of the mixture at 230 nm wavelength. Reduction in the absorption value means the effectiveness of the extract on hydrogen peroxide scavenging.

The CuCl_2_ activity of Chinese yam samples was evaluated using the methodology depicted by Murtaza, Yaqoob, Mubeen, Sameen, Murtaza, Rehman, Alsulami, Korma, Khalifa and Ma [19] with slight modifications. Shortly, a mixture was prepared with 250 μL of CuCl_2_ (0.01 M), 250 μL of ammonium acetate (1 M), 250 μL of neocuproine (7.5 M) and varying quantities of the treatments. The solution was left to stand at room temperature for 30 min and scanned at 450 nm.

The reducing power of the Chinese yam treatments was determined through the modified methodology described in Natić, Dabić, Papetti, Akšić, Ognjanov, Ljubojević and Tešić [20]. Concisely, a mixture was prepared by blending 1 mL of the samples, 50 μL of HCl, 400 μL of FeCl_3_, 400 μL of C_6_N_6_FeK_3_ and 700 μL of H_2_O. The resultant mixture was then incubated at 37°C for 30 min in the dark. Eventually, the absorbance measurements were measured at 720 nm. The results were expressed in mM of ascorbic acid.

### 2.5 Measuring the total sugars and organic acids

To determine the total sugar content of Chinese yam samples, the phenol–sulfuric acid method was used as outlined by Yue, Zhang, Xu, Niu, Lü and Liu [21]. Overall, 0.5 mL of ddH2O and 0.5 mL of 5% phenol solution were mixed with 100 mL of dried ethanol extract. Concentrated H2SO4 was then added in the amount of 2.5 mL. The solution was kept aside for 30 min before measuring the absorption using 490 nm. Pure D-Glucose was used as a control.

The quantification of tartaric, malonic, oxalic, fumaric, succinic, lactic and citric acids was conducted using HPLC according to the method of Büyüktuncel, Kalkan and Şahin [22]. Analysis was done using an Agilent 1100 Series HPLC system that included a degassing unit, quaternary pump, auto sampler (20 μL), and RP-C18 column (particle size = 3 µm, dimensions = 150 × 4.6 mm I.D.) at 25°C with detection being done using a diode array detector. The isocratic eluent used was a 0.01 M/L KH_2_PO_4_ buffer solution (with pH 2.6) using o-phosphoric acid at 0.5 mL/min with wavelength 210 nm.

### 2.6 Color assessment

The color parameters of the sample were analyzed using a HunterLab ColorQuest XE Spectrophotometer (Hunter Associates Laboratory, Virginia, USA) following the CIE color space system, by adopting the methodology of Boateng, Zhang, Li, Saalia and Yang [23]. Parameters that were determined included lightness (L*), redness (a*) and yellowness (b*). Moreover, chroma (C*), hue angle (h°) and total color difference (ΔE) were also calculated to assess the overall color variation.

### 2.7 Micromorphological characterization

Micromorphological properties of the samples were evaluated by utilizing a scanning electron microscope (Phenom™, Thermo Fisher Scientific) at a magnification of × 1000, following the procedure explained by Yaqoob, Liu, Liu, Zheng, Awan, Cai and Liu [24]. The lyophilized specimens were attached to aluminum stubs via double-conducting adhesive tape. To increase conductivity, gold coating was done on the sample. The imaging was carried out under vacuum with accelerating voltage of 5 kV.

### 2.8 Attenuated total reflectance-Fourier transform infrared spectroscopy (ATR-FTIR)

An ATR-FTIR analysis was performed aimed at studying the functional groups of the yam samples utilizing a Nicolet iS50 FTIR spectrometer (Thermo Scientific, Waltham, MA, USA) with an ATR attachment. The methodology of Rehman, Khalifa, Rasheed, Iqbal, Shoaib, Wang, Zhao, Liang, Zhong and Sun [25] was followed for spectral data collection in the 4000–700 cm⁻^1^ range. To determine the element of secondary structure of Chinese yam samples, observed data (amide-I band 1600–1700 cm^−1^) transformation, deconvolution and peak separation were analyzed/ processed applying OMNIC-V32 and PeakFitTM v4.0 Software. The relative percentages of α-helix, β-sheet, β-turn and random coil structures were calculated based on the area under each fitted peak. Furthermore, clarification has been added regarding the origin of the protein signals. The observed changes in protein secondary structure are attributed to both endogenous yam proteins and microbial proteins introduced during fermentation. The peak regions were determined via the use of PeakFitTM v4.0. In addition, the technique of PCA was employed on normalized FTIR spectra (4000–700 cm⁻^1^) via SIMCA v14.1.

### 2.9 X-ray diffraction (XRD) analysis

The crystalline structure of the sample was characterized using X-ray diffraction on a Rigaku AXS Smart Lab diffractometer, following the method of Zou, Li, Su, Wang, Li and Xia [26]. The diffraction analysis was conducted using voltage and current values of 40 kV and 40 mA, respectively, with Cu Kα radiation (λ = 0.15405 nm). The diffractograms were recorded within 2θ values ranging from 3° to 50°, using a scanning speed of 10°/min with steps of 0.02°. The relative crystallinity was determined as a ratio between the area occupied by crystal peaks and the overall diffraction pattern. The relative degree of crystallinity (%) was determined through the peak deconvolution technique, which involves distinguishing crystalline and amorphous regions on diffractograms through Origin 2022 software. The relative crystallinity (%) was found using:

100 Relative Crystallinity (%) = (Ac / (Ac + Aa)) ×

where A_c_ represents the crystalline region and A_a_ is the amorphous region.

### 2.10 Electronic nose analysis

The volatile profile of the Chinese yam sample was evaluated by using a PEN3 electronic nose system (Airsense Analytics GmbH, Schwerin, Germany), following the protocols described by Yu, Huang, Wang, Ren, Zhang and Wang [27]. For each measurement, 10 ml of sample was placed into 50 ml sealed glass vial and was allowed to reach equilibrium. Headspace analysis was done using following settings: measurement time was set at 120 sec, sensor clean time was set at 150 sec, sample pre-incubation time was 5 sec, while internal gas carrier flow rate was 400 mL per min, while injection rate was set at 200 mL per min. Electronic nose used an array of metal oxide semiconductor sensors.

### 2.11 Sensory evaluation

The sensory attributes of the samples were analyzed using quantitative descriptive analysis (QDA) in accordance with the approach by Yu, Huang, Wang, Ren, Zhang and Wang [27]. A panel of 50 untrained food scientists (25 men and 25 women), who ranged in age from 18 to 30 years old, was randomly selected among the students of the School of Food Science and Engineering, Yangzhou University, Yangzhou, China. All panelists were willing to participate in this study. While the participants were untrained, all subjects met the basic qualifications for being a panelist, including not having food allergies, smoking at the time of testing, or any impairments in taste and smell perception Reference No: FOST/DERC/2024/04. In order to reduce any potential bias, the samples were assigned with random three-digit codes. In order to avoid biases during the study, the samples were assigned randomly generated three-digit codes and presented to each panelist in a random order following established sensory testing procedures. The sensory analysis was carried out using the sensory profile which comprised five predetermined attributes namely floral (rose and violet flavors), fruity (mulberry, banana and peach flavors), mellow (alcohol smell), estery (cheesy flavors) and green/grassy (vegetative). This way the previously mentioned attribute of delicate was renamed to green/grassy for better identification purposes. Each attribute was scored using the intensity scale of 0 to 5 whereby zero indicated absence of an attribute while 5 stood for high intensity level. In order to minimize possible carry over effects, participants were asked to cleanse their palate with water after tasting each sample and maintain a standard time interval between consecutive samples. The sensory evaluation was done in one sitting in order to maintain identical environmental conditions throughout testing, namely room temperature, appropriate lighting and minimum noise levels.

### 2.12 Molecular docking

Molecular docking study was done using the latest AutoDock Vina 1.2.0 software. Molecular interaction was done between peptidoglycan of bacteria (PDB ID: 2MTZ) as a macromolecule receptor and quinic acid (QA; PubChem CID: 37439) as a ligand, which were extracted from the protein database and PubChem databases, respectively (https://www.rcsb.org; https://pubchem.ncbi.nlm.nih.gov). Both structures were then optimized by combining fractional charges and energy was minimized using Protonate-3D and MMFF94X force fields and H_2_O was removed, structure refining, energy minimization and 3D protonation Khalifa, Harras and Badawy [28]. The procedure was replicated for the composite compounds and subsequently, 4–5 suitable docked conformations were designed, which were then analyzed based on their hydrophobicity, electrostatic potential, hydrogen bonds and heat map structural dynamics using heatmapper (https://heatmapper.ca/expression). The specific protein–ligand interactions were identified and characterized using the Protein-Ligand Interaction Profiler (PLIP) server (https://plip-tool.biotec.tu-dresden.de/plip-web/plip/index).

### 2.13 Data analysis

The statistical analysis of the experiment was done using the statistical programs IBM SPSS Statistics v.22 and Origin 2022. The variation between samples was assessed through PCA using the program SIMCA v.14.1 using triplicate values of each sample group.

## 3 Results and discussion

### 3.1 Antioxidative polyphenols of Chinese yam

**Fig. 1:**
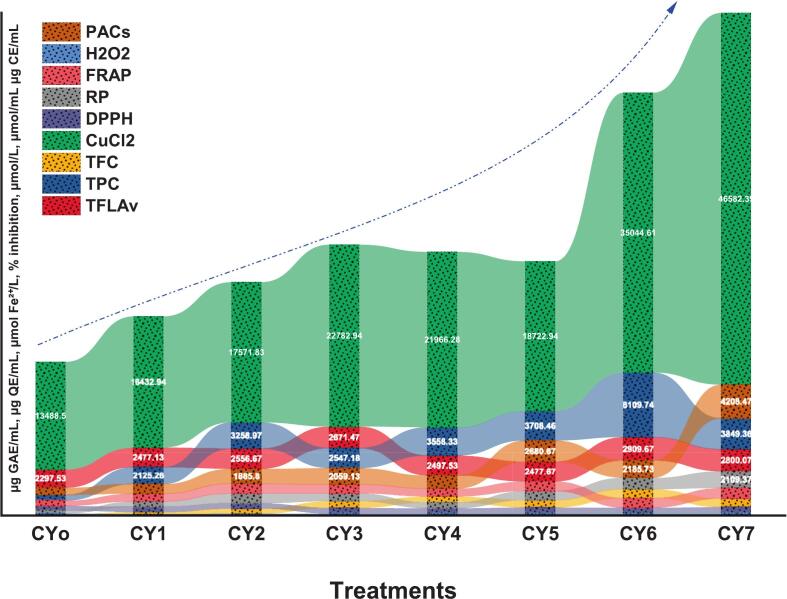
Antioxidant potential of Chinese yam samples (CY_o-_CY_7_) showing TPC, TFC, Tflav, PAC, DPPH, FRAP, H_2_O_2_ scavenging activity, Cu^2+^ reducing capacity and reducing power.

Antioxidant profiling of Chinese yam samples (CY_o_-CY_7_) revealed marked enhancements in bioactive compound content and antioxidant parameters across the treated groups **(**Fig. 1**)**. Statistical analysis was performed using one-way ANOVA followed by Tukey’s post hoc test. Differences were considered significant at p < 0.05. Total flavonoid content showed a significant increase across treatments (p < 0.05), reaching its highest level in CY_6_. Likewise, TPC was found to be elevated, where CY_6_ had the highest TPC value at 8,109.74 µg/mL relative to 689.36 µg/mL in the control group. In addition, T_flav_ attained a maximum value of 1,158.48 µg/mL for CY_6_, almost ten times higher than the control value at 110.67 µg/mL. PAC also was elevated, where it rose from 926.87 µg/mL in CY_o_ to 4,208.47 µg/mL in CY_7_. On the whole, CY_7_ possessed the highest antioxidant capacity, followed by CY_6_ and CY_5_. Nonetheless, although improvements in the bioactive constituents were observed, it must be noted that such results were based on *in vitro* analysis only.

Reduction potential for CuCl_2_ reducing ability, which is used as an indicator of the metal ions' reduction ability, increased from 13,488.50  µM in the control sample (CY_o_) to 46,582.39  µM in CY_7_, indicating the presence of better antioxidant properties. DPPH scavenging ability increased from 574.25% in CY_o_ to 736.37% in CY_7_, implying considerable enhancement of radical neutralization activity (Fig. 1). The value of reducing power (RP) also showed significant improvement, whereby CY_7_ was recorded at 2,109.37  µM while the control sample was recorded at 363.55  µM. FRAP activity showed. Likewise, there was also an enhancement in H_2_O_2_ scavenging ability, in which CY_7_ showed the highest percentage of 284.40% compared to only 128.88% for CY_o_. This clearly indicates the effectiveness of the processes used to increase the antioxidant capability of Chinese yam through polyphenol production.

Despite possessing distinct phytochemical features, studies involving the analysis of its antioxidant mechanism have been rather scarce. Current study is similar to Li, Zhou, Fang, Zheng, Ha and Xu [29], findings highlights the process of ultrasound-assisted extraction technique could increase the quantity of bioactive compound in Chinese yam. In their study, a dramatic increase in total phenolic content (TPC) of about 46% from 63 to 92 mg GAE/g was obtained after conducting the experiment with the help of ultrasonic hydrolysis technique. Besides TPC, there were remarkable variations observed in terms of antioxidant activity especially the reduction in DPPH free radicals. Another study by Ghasemzadeh, Omidvar and Jaafar [30], recorded relatively high levels of TPC and TFC in the leaf extracts of sweet potatoes. Their findings, using DPPH and FRAP tests, indicate that sweet potatoes of the Vardaman variety exhibit powerful antioxidant properties, implying a significant relationship between TPC and antioxidant activity. Similarly, Makori, Mu and Sun [31], reported substantial variation in TPC among different sweet potato cultivars and confirmed that higher TPC was strongly associated with enhanced antioxidant activity.

In contrast, a study by Adomėnienė and Venskutonis [32] incorporation of water yam flour in food formulations resulted in higher TPC and FRAP values; however, there was a smaller increment in the value of TPC compared to the antioxidant activity of the flour. The difference indicates that there are other phytocompounds apart from polyphenols that could significantly contribute to antioxidant activity. In this research, ultrasound treatment followed by fermentation was effective in enhancing the functionality of Chinese yam through improved antioxidant activity. The TFC, TPC, DPPH scavenging activity, FRAP, CuCl_2_ reduction, H_2_O_2_ scavenging activity, RP and PAC were significantly increased especially for samples CY_6_ and CY_7_.

The probiotic species *Lactiplantibacillus plantarum* and *Lactobacillus helveticus* were instrumental in manipulating the antioxidant composition. The enzyme action and microbial interactions helped in the degradation of precursor compounds, resulting in their easy extraction and modification into bioactive compounds. The combined effects of microbial synergism and ultrasound treatment for breaking down cells greatly enhanced the yield of antioxidants from Chinese yam.

**Fig. 2:**
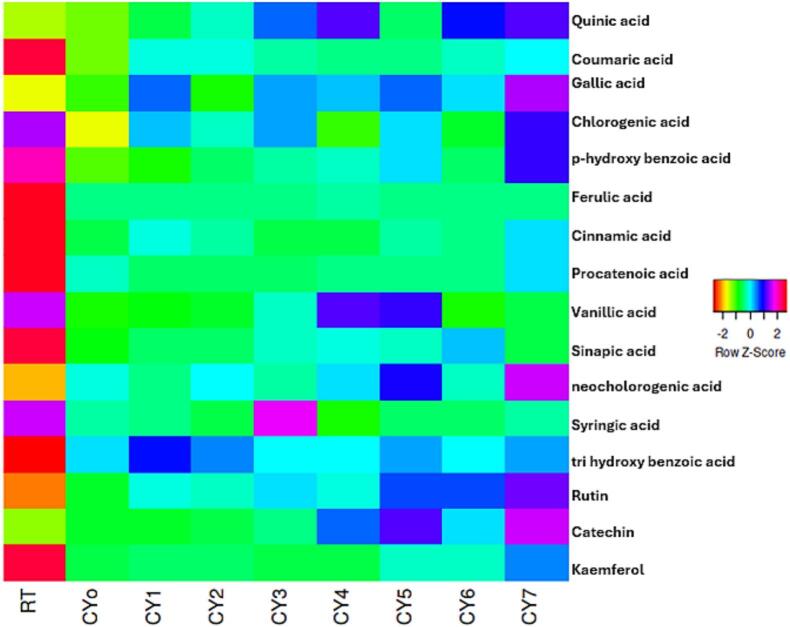
HPLC-based quantification of polyphenolic components in Chinese yam.

Concurrently, HPLC analysis demonstrated that there were notable changes in the polyphenols profile of Chinese yam samples (CY_o_-CY_7_) through the application of ultrasonic waves during fermentation. Out of the total list of compounds analyzed, quinic acid was found to have undergone an almost 12-fold increase, changing from 228.94 to 2,912.46  µg/mL (Fig. 2). Other phenolic compounds also experienced steady and remarkable increases in their concentrations. Gallic acid recorded a rise from 28.63 to 131.43  µg/mL, chlorogenic acid from 6.13 to 13.52  µg/mL and cinnamic acid from 13.13  µg/mL. There were also noticeable increases in the presence of flavonoids, which increased by 2.6 times from 25.58 to 66.62  µg/mL for catechin, doubled from 5.44 to 13.72  µg/mL for kaempferol and grew slightly from 25.21 to 31.99  µg/mL for rutin. Further biologically active compounds include vanillic acid at 14.87  µg/mL for CY4 and sinapic acid at 9.88  µg/mL for CY_6_. Taken together, the data suggest that ultrasound-assisted fermentation in enhancing both the extractability and potential biosynthesis of key nutraceuticals in Chinese yam. This process substantially improves its functional value and health-promoting potential (Fig. 2).

The present findings demonstrate strong consistency with existing research on bioactive moieties enhancement in Chinese yam and other tuber species. The investigation of Xie, Chen, Jiang, Zhou, Guo, Zeng and Zhang [33] observed significant enhancements in the recovery of flavonoids by utilizing ultrasound-assisted extraction for the extraction of polyphenols from the *Dioscorea opposita* starch-tea complex, which led to a tripling of the amount of catechins extracted, with initial percentages of 35.7%. Complementing these results, Yang, Zheng, Yang, Huang, Sun, Zhao, Zhou, Cheong, Wang and Feng [34] elucidated that the fermentation by *Lactobacillus plantarum* of polysaccharides from Chinese yam not only increased total polyphenols by about 7% but also significantly boosted anti-inflammatory properties, validating the role of microbial bioprocessing in improving both quantity and functionality of bioactive compounds derived from tubers. Together, these studies validate our observed patterns of improved phytochemical recovery and bioactivity through advanced processing methods. To conclude, the current study proves that the integration of ultrasound and bacterial strains (*L. plantarum, L. helveticus and L. paracasei*) is highly efficient in optimizing the bioactivity of Chinese yam via three major processes: I) increased phenolic release; II) improved bio-conversion of complex compounds III) activation of organic acid biosynthesis. The application of this innovative combination of physical and microbiological treatment significantly improves the bioactive value of Chinese yam by maximizing the concentration and availability of its bioactive substances. Despite differences between Chinese yam (*Dioscorea opposita*) and sea buckthorn (*Hippophae rhamnoides*) both plants share high concentrations of polyphenols and have been widely used in functional food and nutracetucial foods can be used as a ingredient. Sea buckthorn has been extensively examined for its polyphenol content and distribution in different processing and growth conditions. In view of the paucity of data on polyphenolic content of Chinese yam subjected to fermentation and ultrasonic treatment, the information about sea buckthorn may be regarded as an important biochemical reference for interpreting the dynamics of flavonoids and antioxidants.

### 3.2 Total sugars and organic acids of Chinese yam

**Fig. 3:**
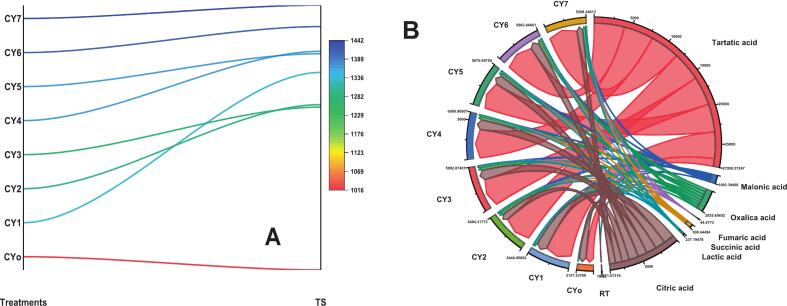
(A) Total sugar content of Chinese yam treatments (CY_o-_CY_7_). (B) Organic acid profiles of Chinese yam samples after fermentation.

The overall amount of sugar found in the Chinese yam specimens also varied considerably depending on the treatment applied (Fig. 3A). The control group without fermentation (CY_o_) had the lowest sugar level of 1,017.81 mg glucose equivalent/gram (mg GE/g) that served as the standard value against which other values would be compared. On the other hand, all samples with ultrasound-assisted fermentation (CY_1_-CY_7_) showed an increased sugar content, with the highest being in CY_7_ at 1,441.32 mg GE/g. The increase in total sugars is attributed to ultrasound-enhanced release and enzymatic hydrolysis of complex carbohydrates, which may exceed microbial sugar utilization during early fermentation stages.

A progressive increase in total sugar content was observed across the treatment series, from CY_1_ (1,333.81  mg GE/g) to CY_7_ (1,441.32  mg GE/g), with minor fluctuations among intermediate samples. The highest sugar concentrations were recorded in CY_6_ (1,407.19  mg GE/g) and CY_7_, suggesting that these specific treatment conditions were most effective in promoting sugar accumulation. The noted increase is attributed to the combined effect of ultrasound and microbial enzymatic activity, which improves the breakdown of complex carbohydrates such as polysaccharides and oligosaccharides into simpler and measurable sugars. Ultrasound treatment facilitates cell wall disruption and enhances substrate accessibility, thereby accelerating the release of bound carbohydrates. Simultaneously, microbial enzymes such as amylases and cellulases hydrolyze complex structures into reducing sugars. It is therefore likely that the rate of sugar release exceeded the rate of sugar utilization by lactic acid bacteria during the initial stages of fermentation for 24 h, resulting in a net increase in measurable total sugars. Moreover, the method used for sugar quantification (e.g., phenol–sulfuric acid method) measures total soluble sugars, including newly released hydrolysis products, which may further contribute to the substantial increase.

The present results are parallel with the findings of Ramón, Taschetto, Lunelli, Mezadri, Souza, Foletto, Jahn, Kuhn and Mazutti [35] who showed that combining ultrasound with enzymatic hydrolysis improved the fermentable sugar yields in yam from 0.32 g/g to 0.42 g/g of dry mass material, further emphasizing how the physical techniques can synergize with enzymatic processes to optimize the recovery of sugar. While some investigation enhances the sugar contents through enzymatic treatments, contrasting findings were observed by Chen, Zhu, Niu, Song, Zhang, Chen and Chen [36] during the fermentation of yam juice with *L. plantarum* and *Streptococcus thermophilus*. Their report documented a substantial decrease in sugar content, exhibiting that these bacterial strains actively metabolize the available sugars during fermentation. The differences in findings show the significant role that processing methods and microbial strains have on the composition of sugars obtained from the fermentation process of yam. The decrease in sugar content indicates potential uses, such as producing low sugar drinks, through the appropriate choice of microbes and processing methods. In contrast, research employing the strains of *L. plantarum, L. helveticus* and *L. paracasei* showed a high increase in sugar content from Chinese yam (as indicated by the increase in sugar content from CY_o_ to CY_7_, particularly CY_6_/CY_7_).

The quantification of organic acids using Chinese yam demonstrated substantial variations in acid concentrations among different sampled, with all values reported in µg/mL (Fig. 3**B**). Citric acid emerged as the predominant organic acid, reaching its highest concentration of 2912.46 µg/mL in the CY_7_, revealing its role as a key metabolic intermediate in yam tubers. Lactic acid showed the most dramatic increase during fermentation, rising from 228.94 µg/mL in CY_o_ to 1856.72 µg/mL in CY_6_, clearly implying active microbial conversion by the lactic acid bacteria strains. Succinic acid exhibited a notable peak of 1024.58 µg/mL in CY_5_, showing enhanced activity in the tricarboxylic acid cycle under ultrasound-assisted fermentation conditions. The concentrations of tartaric acid (ranging from 286.39 to 324.75 µg/mL) and malonic acid (between 198.47 and 225.63 µg/mL) remained stable among all treatments, showing minimal degradation despite the fermentation process. The amount of fumaric acid also increased significantly from 86.29 µg/mL in CY_o_ to 172.54 µg/mL in CY_4_ because of changes in the mitochondrial metabolism. In contrast, there was a decline in oxalic acid content by around 40%, from 156.42 µg/mL in CY_o_ to 93.85 µg/mL in CY_7_, likely because of conversion by microbes (Fig. 3B).

The present findings are consistent with the study of Batista, Ramos, de Figueiredo Vilela, Dias and Schwan [37], who investigated the fermentation of yam using indigenous LAB strains, including *Leuconostoc lactis* and *Lactobacillus plantarum*. The researchers observed a significant production of lactic acid, reaching concentrations around 8 g/L within 6 h of fermentation. This increase in lactic acid was associated with a decrease in pH, enhancing the microbiological stability and flavor of the fermented yam. Additionally, the study noted a reduction in oxalate content, attributed to the pre-cooking process before fermentation. In another study, Cui, Zhou, Li, Wang, Li and Chen [38] demonstrated that Chinese yam substrates, when subjected to *in vitro* fermentation, significantly enhanced the production of microbial metabolites such as lactic acid and short-chain fatty acids, supporting gut health and improving metabolic activity. Lactic acid build-up and medium acidification were observed, which corroborate previous findings on increased amounts of citric and lactic acid found in the present experiment. Fermentation is known to increase some organic acids but decrease others. Ultrasound fermentation has caused dramatic changes in the acid composition of Chinese yam, resulting in an increased level of useful citric acid by 12 times and lactic acid more than eight times, while the level of harmful oxalic acid has decreased.

### 3.3 Color analysis of Chinese yam

**Fig. 4:**
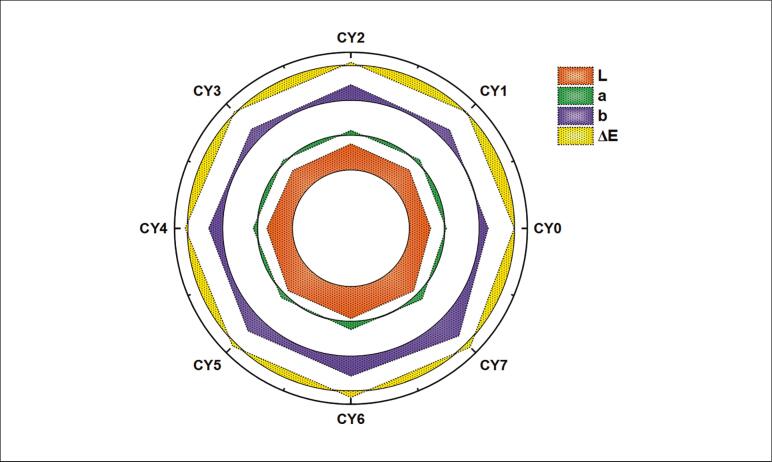
Colorimetric analysis (L, a*, b* and ΔE values) of fermented Chinese yam samples.

The color assessment of Chinese yam samples (CY_o_ to CY_7_) revealed significant changes in the L*, a*, b* and ΔE values (Fig. 4), indicating noticeable differences in appearance among the treatments. The lightness (L*) value of CY_o_ was 65.41 ± 1.31, whereas CY_6_ exhibited a much higher lightness of 92.69 ± 1.85. This indicates that CY_6_ was the brightest sample, while CY_o_ appeared the darkest. The redness-greenness parameter (a*) increased from 16.6 ± 0.33 in CY_o_ to 32.55 ± 0.65 in CY_6_, with CY_6_ showing a more pronounced red hue. Similarly, the yellowness-blueness component (b*) rose from 34.50 ± 0.69 in CY_o_ to a peak of 73.50 ± 1.47 in CY_7_, suggesting that CY_7_ exhibited the most intense yellow coloration among all ultrasound-assisted fermented samples. The total color difference (ΔE), which reflects the overall deviation from the reference color CY_o_, ranged from a low of 12.55 ± 0.25 to a high of 28.20 ± 0.56 in CY_6_, indicating significant color variation. It was evident from CY_5_, CY_6_, and CY_7_ that there were increased L*, a* and b* values, implying that the processed samples were relatively lighter, redder, and yellower than the rest. This clearly indicates that the processing conditions used for CY_5_ to CY_7_ influenced the appearance of Chinese yam.

Our present results about the color enhancement of Chinese yam fermented with ultrasound assistance are consistent with the study of Wu, Li, Zeng, Cai, Teng, Wu, Sun and Bai [39], who revealed that ultrasound treatment enhances the color parameters in Sanhua plum wine, with increases in a* and b* values. The findings attributed this to the deactivation of polyphenol oxidase and lowered anthocyanin degradation, techniques that could likewise apply to Chinese yam. Similarly, Li, Shao, Hao, Kang, Wang, Zhu, Zhao, Shi, Lu and Yi [40] demonstrated that fermentation of Chinese yam by *Saccharomyces boulardii* led to substantial metabolic variations, comprising increased polyphenols and flavonoids content. An increment in L, a, b and ΔE values suggests a rise in lightness, vividness and visual attributes when contrasted with non-fermented samples. The reason for such an improvement could be due to ultrasonication, which aids in the preservation of pigments and hinders browning reactions, possibly through mass transfer and cellular disruption.

### 3.4 Micromorphological characterization of Chinese yam

**Fig. 5:**
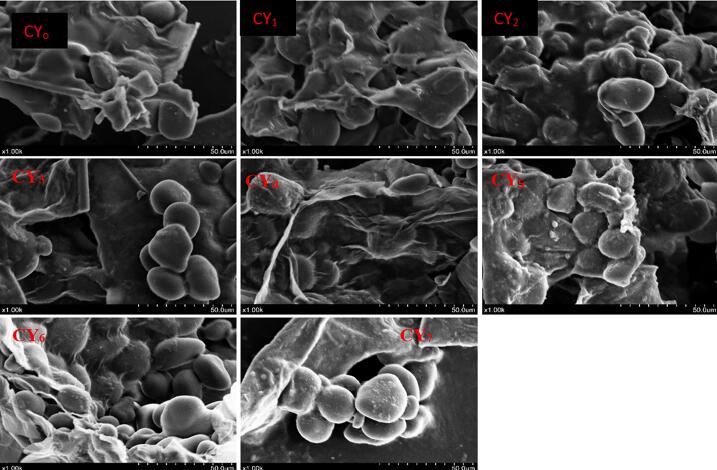
SEM pictographs showing microstructural changes in Chinese yam samples (CY_o_-CY_7_).

SEM images of Chinese yam treatments (CY_o_-CY_7_) revealed notable differences in surface morphology under various processing conditions (Fig. 5). The CY_o_ displayed a smooth and compact surface with intact cell wall structures, characteristic of the native tuber. In contrast, CY_1_-CY_7_ exhibited porous, fractured surfaces indicative of structural breakdown. It is provoked by the physical action of ultrasound, which leads to cavitation (the formation and bubble collapse in the medium), producing shear forces that are extremely intense. High travelling speeds along with pressure pulsations, on equilaterally produce mechanical ruptures and fragmentation into cell walls by these forces. Furthermore, primary oxidative radicals produced by sonication may chemically alter polysaccharide structures as well leading to microstructural changes observed. The compact and smooth microstructure observed for native Chinese yam is consistent with previous reports noting the low surface roughness and tightly packed cellular arrangements in untreated tuber cell walls. The samples showed considerable damage to their surfaces, which was attributed to porosity and cracking, caused by the dual effect of amylase enzyme hydrolysis and physical methods. Such changes are desirable because they increase the ability of the samples to absorb moisture and swell [41]. Our present findings share notable similarities with the work of Liu, Liu, Fan, Qin and Wang [42], who investigated the effects of different drying pretreatments on Chinese yam starch. Their SEM analysis revealed that while balmy air drying and subcritical dimethyl ether dehydration caused surface indentations and structural disruptions, freeze-drying preserved granule integrity. These observations support our results by demonstrating that the microstructure of Chinese yam, as visualized by SEM, can be significantly altered by both enzymatic and physical treatments. Similarly, Murtaza, Yaqoob, Mubeen, Sameen, Murtaza, Rehman, Alsulami, Korma, Khalifa and Ma [19] studied the combined effects of fermentation and triple-frequency ultrasound-assisted lactic acid bacteria fermentation (*L. helveticus and L. plantarum*) over 24 and 48 h on rice lees. SEM showed that there was a marked difference in the microstructure of Chinese yam when subjected to treatment; untreated samples were smooth and dense, whereas treated samples were porous and broken. The effect of enzymatic action and ultrasound on Chinese yam improved its properties such as swelling, hydration, and digestibility.

### 3.5 FTIR, XRD, secondary structure and theoretical binding analysis within the Chinese yam

**Fig. 6:**
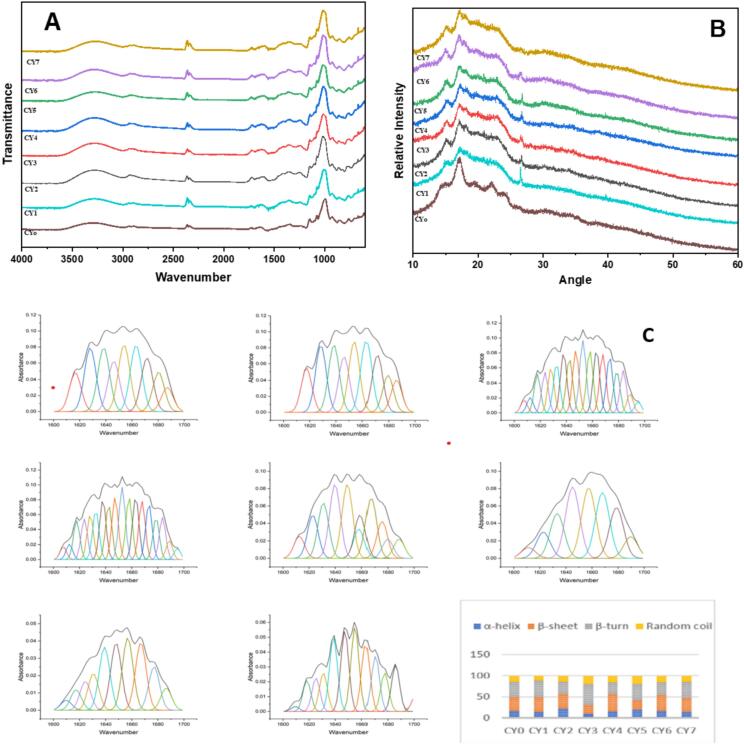
(A) FTIR spectra revealing functional group shifts among Chinese yam samples, (B) XRD analysis showing reduced crystallinity in treated Chinese yam samples (C) Peak Fit Analysis of protein secondary structures in Chinese yam samples.

FTIR spectroscopy of Chinese yam treatments (CY_o_-CY_7_) revealed distinct absorption peaks indicative of biochemical changes induced by processing (Fig. 6**A**). An absorption band ranging from 3500 to 3200 cm⁻^1^ was a result of O–H bond vibrations related to polysaccharides and water molecules along with N–H vibrations linked with proteins and amino acids. Absorption bands in the region of 3000 to 2800 cm⁻^1^ represented C–H bond vibrations related to aliphatic chains of lipids and carbohydrates. An absorption band between 1740 to 1700 cm⁻^1^ showed C=O bonds present in esters or organic acids, such as pectin. Absorption bands between 1650 to 1550 cm⁻^1^ represented amide I and II bands indicating presence of proteins in the sample. Absorption bands ranging from 1200 to 1000 cm⁻^1^ indicated C-O-C and C-OH bond vibrations that occurred in biopolymers like starch and cellulose. The CY_o_ displayed baseline peaks consistent with native Chinese yam composition, while treatments CY_1_ through CY_7_ showed variations in peak intensities, reflecting structural modifications due to processing. Notably, CY_5_ exhibited a pronounced C=O peak, suggesting enhanced esterification of pectin, whereas CY_3_ showed intensified amide bands, indicative of higher protein retention. These spectral shifts suggest that specific treatments can alter the functional properties of Chinese yam, potentially impacting its bioactivity and nutritional value. Overall, the FTIR spectral analysis provides valuable insights into molecular modifications induced by processing, with important implications for the development of optimized yam-based functional products. The present findings align with the research of Yang WeiFang, Wang Ying, Li XiuPing and Yu Ping [43], who employed FTIR to identify characteristic polysaccharide absorption peaks, including broad O–H, C–H and C–O–C bands. Their study also detected protein-related amide group peaks, consistent with our observations. Another study of Yang, Wang, Li and Yu [44], reported notable changes in the 1100–1300 cm⁻^1^ region, corresponding to C–O–C and C–O–H stretching vibrations of cellulose and starch. However, these results contrast with those of Yang, Zheng, Yang, Huang, Sun, Zhao, Zhou, Cheong, Wang and Feng [34], who reported no significant changes in the major functional group areas (C–H, O–H, and C-O) prior to and following fermentation by L. plantarum, indicating that the fermentation process did not result in structural modifications of the basic polysaccharide structure. Overall, it can be seen that both *L. plantarum, L. helveticus* and *L. paracasei* enzymatically modify Chinese yam, thereby improving its nutritional value.

Analysis of Chinese yam starch samples, including CY_1_, using XRD revealed notable changes in crystallinity (Fig. 6**B**). Most strikingly, CY_1_ exhibited broader and lower-intensity diffraction peaks, particularly in the 2θ range of 15 to 25°, compared to CY_o_. The decrease in the crystallinity is due to the disruption of the starch crystalline zones through ultrasonic cavitation and hydrolysis by enzymes during fermentation process. Despite the unchanged positions of the typical diffraction peaks at 2θ, suggesting preservation of the type of the crystals, the decrease in the peak intensities and crystallicities demonstrates the destruction of the crystal structure. The above results provide quantitative evidence for the previous qualitative observations regarding the destruction of the crystals. This suggests that ultrasound treatment leads to notable destruction in crystalline content, by collapsing the crystalline portions of granules during cavitation effects produced during ultrasonic waves. Due to the disruption of amorphous regions by cavitation and shear forces, XRD analysis shows that crystallinity decreases (such as less intense peaks in CY_1_). Although peak positions are constant that is crystalline structure is preserved, relative crystallinity between samples varies sharply. The resulting structural changes improve functional traits including solubility and digestion rate, illustrating the effectiveness of ultrasound to modify starch properties.The present findings are corroborated by the research of Prakruthi, Krishnan, Medha, Kumarakuru, Kumari, Surekha, Alavilli, Kaushik, Hashem and Alotaibi [45], who reported that ultrasound-assisted samples reduced the strength of distinctive diffraction peaks of Chinese yam starch, resulting in a decrease in crystallinity while maintaining the overall crystalline type. This proved that ultrasonic cavitation largely affects the amorphous parts of starch granules, causing morphological changes but not affecting the core crystalline structure. In another study of Xie, Chen, Jiang, Zhou, Guo, Zeng and Zhang [33], the complexation of Chinese yam starch with tea polyphenols resulted in a B^+^V^-^type crystalline structure, as signified by XRD analysis. Starch-polyphenol interactions enhanced thermal stability and altered crystallinity. Ultrasound treatment of the starch from the Chinese yam decreased the crystallinity of the starch by destroying the amorphous areas without altering the nature of the crystalline starch. Bacterial culture (*Lactobacillus plantarum, L. helveticus, L. paracasei*) also altered the physicochemical characteristics of starch by manipulating starch structure/crystallinity.

**Fig. 7:**
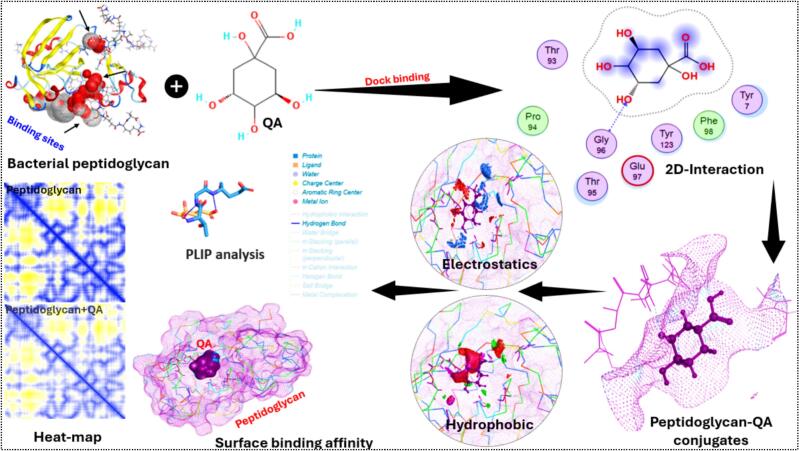
Molecular docking and interaction analysis of quinic acid (QA) with bacterial peptidoglycan of Chinese yam samples.

Due to the changes that happened in the FTIR and XRD patterns, which might be because of the interaction between the bacterial proteins and polyphenols of Chinese yam, we decided to do molecular docking to theoretically discover this interaction. The primary objective of molecular docking was to provide molecular-level insights into the interaction between quinic acid (QA), peptidoglycan and to support the experimentally noted structural alterations in the fermented product. Fig. 7 comprehensively illustrates the molecular interplay between peptidoglycan and QA, employing multiple analytical techniques to dissect binding dynamics and affinity. The binding score of −5.13 and binding affinity of −2.54 kcal/mol exhibits a moderately stable interaction between QA and peptidoglycan. Hydrogen bonding interactions were observed, particularly involving Gly96, which contributes to structural stability of the complex. Hydrophobic interactions with residues such as Thr95 and Tyr7 further support the stabilization of the ligand–protein complex. Moreover, electrostatic interactions involving multiple residues were identified, revealing their role in binding specificity and strength. These interactions may explain the structural modifications noted in FTIR and XRD analyses, as the formation of polyphenol-protein complexes can influence molecular organization and crystallinity. The formation of such complexes may contribute to the stabilization of polyphenolic components during fermentation, potentially improving their functional parameters including antimicrobial and antioxidant potential. However, these outcomes are based on *in silico* analysis and serve as supportive evidence only. Further experimental validation is required to confirm the biological and functional implications of these interactions.

The analysis of secondary protein structures in Chinese yam samples (CY_o_-CY_7_) revealed significant variations in protein conformation as a result of different processing treatments in Fig. 6**C**. The unfermented control sample, CY_o_, exhibited a balanced distribution of structural elements, comprising 16.76% α-helix, 32.49% β-sheet, 36.27% β-turn and 14.48% random coil indicative of a stable, native protein conformation. Among the processed samples, CY_2_ demonstrated the most ordered structure, with the highest α-helix content (21.70%), suggesting enhanced thermal stability and preservation of native secondary structures. In stark contrast, CY_3_ underwent the most pronounced structural transformation, with α-helix content dropping to 10.32% and β-sheet to 20.32%, while β-turn increased to 50.16% and random coil to 19.20%. These alterations suggest extensive protein denaturation, likely induced by processing-induced stress. CY_4_ exhibited the highest β-sheet content (40.82%), which may reflect increased protein aggregation or crystallinity. Both CY_1_ and CY_5_ showed elevated levels of β-turns and random coils, indicating enhanced protein flexibility and partial unfolding; CY_5_ in particular displayed a mixed conformation, with 19.58% α-helix and 19.77% random coil. CY_6_ retained relatively high β-sheet content (36.78%) alongside moderate levels of other structures, suggesting structural rearrangement without complete denaturation. These results have proved beyond doubt that different processing methods greatly influence the structure of Chinese yam proteins. There are specific processes, for example, CY_3_ and CY_5_ which cause significant denaturation, whereas there are other processes such as CY_2_ that help preserve the structure of the proteins.

The structural transitions observed in this study are consistent with findings from prior Fourier-transform infrared (FTIR) spectroscopy research. For instance, Shao, Jiao, Wu, Xu, Li, Jiang, Zhang and Mao [46] conducted an in-depth analysis of Dioscorea opposita protein structures, reporting comparable secondary structure distributions α-helix (18–23%), β-sheet (30–38%) and random coil (13–17%) which align closely with our baseline observations. In contrast to our outcomes, Dong, Li, Zhang, Liu and Ren [47] investigated flaxseed protein isolates and found significantly different behavior. Their results indicated high thermal stability, with only minimal changes in secondary structure following thermal treatment: α-helix content decreased slightly from 35.9% to 34.7% and β-sheet content increased marginally from 38.2% to 39.0%. This suggests that flaxseed proteins possess a more rigid and heat-resistant conformation compared to the thermally sensitive proteins of Chinese yam. Notably, this study employed specific probiotic strains: *Lactobacillus plantarum*, *L. helveticus and L. paracasei* for fermentation, which contributed to conformational changes in the yam proteins. However, fermented materials had a decrease in α-helices and an increase in β-turns and random coils, showing that there was an unfolding effect in proteins due to microbial enzymes.

### 3.6 Electronic nose of Chinese yam

**Fig. 8:**
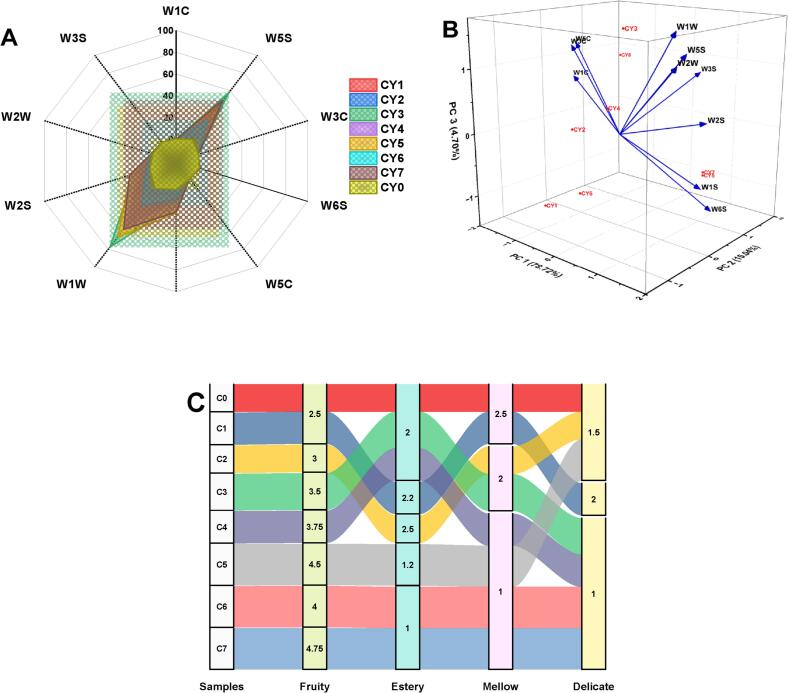
(A) Electronic nose sensor response radar graph for volatile compounds in Chinese yam samples. (B) Principal component analysis of E-nose data, differentiation among treatments. (C) Sensory profiling of aroma descriptors in Chinese yam samples.

The radar plot showing the variations in responses of the E-nose towards the treatments on Chinese yams (CY_o_-CY_7_) shows the sensitivity of each sensor type (W1C, W5S, W3C, W6S, W5C, W1S, W1W, W2S, W2W and W3S) towards unique classes of volatile compounds (Fig. 8A-B). The CY_o_ exhibited the lowest overall sensor responses, indicating a low concentration of volatile substances. In contrast, CY_6_ and CY_7_ demonstrated strong and diverse sensor responses, with pronounced activity at sensors W1C (aromatics), W5S (nitrogen oxides) and W1S (methane and broad-range volatiles), reflecting a complex and intensified volatile profile. Samples CY_5_, CY_6 and_ CY_7_ showed intense and widespread sensor activity, suggesting substantial emission of aroma-active volatiles. Moderate sensor responses were observed in CY_2_ and CY_4_, while CY_1_ and CY_3_ exhibited dynamic but comparatively lower activity than the other fermented samples, though still higher than the control. Overall, the volatile emission profiles of Chinese yam samples varied significantly as revealed by E-nose analysis. These differences result from the use of different co-culture bacterial strains or processing methods, which influence the production and release of aroma-active volatile compounds. The present results corroborate earlier findings by Pan, Zhang, Bai, Liu, Zhang, Tian, Zhou, Zhou, Liao and Hou [48], who demonstrated that different microbial strains substantially influence the volatile compound profile during sweet potato fermentation. For instance, fermentation using *S. cerevisiae* and *L. plantarum* resulted in increased alcohol and ester production, contributing to increased volatiles, an outcome that is in agreement with our results on the use of different co-cultured bacteria for Chinese yam. Similarly, the study of Rajendran, Silcock and Bremer [49] reported elevated levels of esters, alcohols and acids in yam juice fermented by *L. plantarum* and *S. thermophilus*, which contributed to enhanced aroma profiles. Fermentation by microorganisms played an important role in improving the aroma characteristics of the Chinese yam. The presence of more floral aroma components such as 2-phenylethyl alcohol and results of electronic nose analysis indicated the increase in the complexity of the aroma owing to higher responses towards nitrogen oxides and volatiles. Lactic acid bacteria played an important role in producing volatiles.

### 3.7 Sensory profiling of Chinese yam

The sensory characteristics of Chinese yam samples (CY_o_-CY_7_) exhibited noticeable differences across five attributes: estery, fruity, mellow, delicate and floral. Among these, the floral and fruity notes were most enhanced in CY_5_, CY_6_ and CY_7_, where scores reached as high as 4.5 (**Fig. C**). This indicates a substantial increase in aroma intensity and complexity in these treatments. Notably, CY_7_ displayed strong fruity and floral attributes, suggesting that the processing significantly influenced the release or production of volatile aromatic compounds. In contrast, the mellow, estery and delicate characteristics remained consistently low across all samples, scoring below 2.5. The delicate attribute was particularly subdued, with values around or below 1.0 in samples CY_3_ through CY_7_, implying that the treatments favored the development of more pronounced rather than subtle aromatic notes. In a similar way, the mellow attributes were linked with smoothness and balance. This decreased in the fermented samples when compared with the control yeast, which showed high values for estery and mellow. On the whole, this indicated that the effect of ultrasound on fermentation resulted in the enhancement of some flavor profiles and the suppression of others. The present outcomes are somewhat contrary reported by Yu, Li, Tao, Zhao, Liu and Cui [50], the occurrence of esters and alcohols like ethyl acetate and hexanol in raw yam, which are responsible for its sweet, fruity smell. These volatiles are characteristic of fresh tubers and are particularly noticeable in minimally processed yams. The present findings align with the prior study of Yaqoob, Imtiaz, Awan, Murtaza, Mubeen, Yinka, Boasiako, Alsulami, Rehman and Khalifa [51] on fermented mulberry juice, the primary flavor attributes were delicate, fruity, flowery, mellow and estery. Each descriptive characteristic's intensity was quantified, demonstrating the descriptors' applicability in sensory profiling of plant-based diets. The results of sensory profiling revealed that co-culture fermentation enhanced the flavor attribute, with considerable changes in floral and fruity notes. A study of Aguiar, Freitas Júnior, Andrade and Almeida [52] agrarian students on the differences between two species of yams found variations in their sensory properties, such as taste and odor, demonstrating that sensory evaluation is an important factor in consumer selection of yam products. The use of fermentation and ultrasound improved the fruity/ floral scent in the Chinese yam, while reducing its mild nature, probably through microbiological conversion and ultrasound-induced cell rupture.

## 4 Conclusion

This investigation reveals that the integration of multi-frequency ultrasonication with lactic acid bacteria co-culture fermentation is an effective strategy to improve the functional and structural characterization of Chinese yam. Beyond reporting enhancement in bioactive moieties, this study provides novel insight into the synergistic mechanism between acoustic cavitation and microbial metabolism, emphasizing how ultrasound-induced cell disruption improves substrate accessibility and promotes microbial bioconversion of bound phytochemicals. A key scientific contribution of this research lies in establishing a cyclic fermentation ultrasonication fermentation framework, which offers a controlled approach for maximizing phenolic release, structural changes and sensory profiling in plant-based matrices. Despite these advancements, certain limitations should be acknowledged. The study is primarily based on *in vitro* analyses and the bioavailability and physiological relevance of the released bioactive components were not validated *in vivo*. Moreover, process scalability and energy efficiency of multi-frequency ultrasonication remain to be evaluated for industrial applications. Additionaly, the interaction mechanisms between specific microbial strains and ultrasound conditions require deeper molecular-level investigation to fully elucidate their synergistic effects. Future research should therefore focus on *in vivo* validation, optimization for industrial-scale processing and techno-economic assessment. Exploration of additional probiotic strains, tailored ultrasound parameters and advanced delivery systems such as encapsulation may further enhance the applicability of this approach. In a nutshell, this study contributes to the advancement of sustainable food processing technologies by providing a mechanistic and process-oriented framework for boosting the functional quality of plant-based foods.

### Ethics statement

No animal and human experiments. The sensory study was performed in a safe and hygienic food production environment, with the generous assistance of the School of Food Science and Engineering at Yangzhou University, China.

## CRediT authorship contribution statement

**Aysha Imtiaz:** Writing – original draft, Methodology, Investigation, Formal analysis, Data curation, Conceptualization. **Sanabil Yaqoob:** Writing – original draft, Methodology, Investigation, Formal analysis, Data curation, Conceptualization. **Kanza Aziz Awan:** Writing – review & editing, Software, Methodology, Data curation. **Hiba Naveed:** Writing – review & editing, Software, Methodology, Data curation. **Ahmad Faraz:** Writing – review & editing, Methodology, Formal analysis, Data curation. **Waleed Sultan:** Writing – review & editing, Visualization, Software, Data curation. **Remah Sobhy:** Writing – review & editing, Methodology, Investigation, Formal analysis. **Akmal Nazir:** Writing – review & editing, Validation, Investigation, Funding acquisition. **Jian-Ya Qian:** Writing – review & editing, supervision, Methodology, Data curation. **Qing Shen:** Writing – original draft, Supervision, Software, Resources, Project administration, Funding acquisition, Conceptualization.

## Declaration of competing interest

The authors declare that they have no known competing financial interests or personal relationships that could have appeared to influence the work reported in this paper.
